# New York Letter

**Published:** 1897-05

**Authors:** Ogden C. Ludlow

**Affiliations:** 2309 Seventh Avenue; New York


### *CORRESPONDENCE.*

### NEW YORK LETTER.

NEW YORK, April 10, 1897.

The recent action of our Health Board in placing tuberculosis on the list of those diseases which physicians are compelled to report to the board, has stirred up a good deal of criticism—some adverse and a good deal favorable. It is really no new thing, as for the past three years our hospitals and public institutions have been required to notify the board of their cases of tuberculosis. Those who object to the new order do so on the ground that it is likely to entail needless misery and infringe too much on personal rights. On the other hand, it is asserted that the object of this latest move is to give an opportunity to push more vigorously than has been possible in the past the campaign of education, without which the sanitarian can hope to accomplish but little in the way of controlling this dread disease. In this connection the New York *Herald* makes the following very pertinent comment: "In large cities physicians are required to give notice to the health authorities of cases of certain infectious diseases, the mortality of which is small compared with the mortality of consumption. There is every reason, therefore, why the New York Board of Health should be vigorously sustained in its endeavor to arrest the spread of tuberculosis."

*Apropos* of this subject, I would like to refer to a discussion at the Academy of Medicine on the treatment of pulmonary tuberculosis. On this occasion Dr. S. A. Knopf, after setting forth the advantages of special sanatoriums, and describing the best method of constructing them, asserted that the experience in large European sanatoriums for pulmonary tuberculosis had shown that the running expenses were scarcely any higher than those of any large general hospital. A point that he emphasized, and one which is not uncommonly overlooked by the physician, is that the digestive powers of these patients is often far in excess of what might be supposed from the poor appetite. They should take daily eight or ten glasses of milk, sipping it slowly. It is also well to remember that consumptives often unnecessarily rack and exhaust themselves by coughing, purely as a result of habit, and when there is nothing to expectorate.

Not many years ago the treatment of pulmonary tuberculosis was a fit theme for the pen of the pessimist; to-day the observations of both pathologists and clinicians give us a reason for the faith that is in us, and spur us on to new conquests in curative as well as in preventive medicine. Listen to the words of Dr. E. L. Trudeau, the director of the well known Adirondack Sanatorium: "Apparently, 20 per cent. of our patients are permanently cured, and in about 30 per cent. more the disease is arrested." In the discussion already referred to, Dr. Trudeau stated that the practitioner should be on his guard, lest he be misled by an apparently robust development of the chest into the belief that such a physique was incompatible with the occurrence of pulmonary tuberculosis. In many cases, slight hemoptysis is the first sign of the onset of this disease, but whenever the physician notes a slight impairment of the appetite, associated with an increase in the pulse rate, occasional slight fever, pallor in the morning and slight cough, his suspicions should be aroused sufficiently to lead him to make a searching investigation. Dr. Trudeau is one of the few who still believe in the use of tuberculin in the human subject. He says that its diagnostic value is not appreciated, and that when the initial dose does not exceed half a milligram, he had never known it to cause serious constitutional disturbance. Another diagnostic test that may be resorted to in the very early stage is the injection of the sputum into a guinea-pig. If careful physical examination, aided by these tests, fails to establish the presence of pulmonary tuberculosis, we may feel reasonably sure that it does not exist. While speaking of the use of tuberculin in this diagnostic way, it may be interesting to note also that this observer has used it therapeutically during the past four years. While it has seemed to be harmful in the active stage of the disease, its use is unattended with danger in the apyretic cases. Its beneficial action is most apparent in preventing relapse. As to the matter of climate, he does not believe that there is any specific climate for tuberculosis, but certain climates are more favorable than others. Thus, the colder and more tonic climates are well suited for the incipient cases. These patients should live out-doors most of the time, although, in addition to being warmly clad, they should be sheltered from the winds. One of the most serious, and at the same time commonest, mistakes made by the physician is to insist that the consumptive shall take active exercise in the open air. It should be borne in mind that what is but moderate exercise to a person in health may prove disastrous and exhaustive to the consumptive. Where there is any fever active exercise must be avoided—indeed, it is better to err on the side of being too cautious in prescribing active exercise. Dr. Hermann M. Biggs stated that so far as his experience had gone, he had been much pleased with tuberculin as a diagnostic test, and Professor Koch had informed him that in the five thousand cases in which he had employed this agent, there had not been a single mishap.

One evening here was recently devoted to discussion of pleurisy—chiefly pleurisy with effusion. Dr. R. C. M. Page, who opened the discussion, was of the opinion that the curved line of dullness is a sign of no diagnostic value in differentiating between consolidated lung and pleurisy with effusion. On the other hand, Dr. E. G. Janeway and Dr. Egbert LeFevre believed it to be a useful sign. Dr. Le Fevre explained that the curved line of Ellis being formed in proportion to the resilience of the lung, it afforded us a guide to the condition of the lung. For instance, if the lung were so bound down that this line was not formed, we would be led to infer that the pleurisy was of secondary origin. As to the performance of thoracentesis, Dr. Page thought we should not resort to this measure so long as the case was progressing favorably, as it would then tend to increase the inflammation, and the tendency to thickening of the pleura. Where the respiration is seriously embarrassed, or the pleural cavity remains half full of fluid for several weeks without effort at absorption, he would aspirate in the axillary line, at the fourth intercostal space on the right side and the fifth on the left. Dr. Janeway would aspirate whenever the dyspnea was sufficient to make the patient uncomfortable. He preferred to do it in the fifth or sixth interspace in the axillary line, or between the eighth and ninth ribs in the scapular line. He thought it would often be unnecessary to resort to aspiration, if absorption of the fluid were promoted by limiting the quantity of fluid ingested by the patient to about two-thirds the amount of urine voided, and at the same time giving the patient salt or iodide of potassium to make the blood "thirsty." Blisters are chiefly useful in the later stages. Dr. Francis Delafield said that within the last few years he had come to look upon thoracentesis as *the* treatment for pleurisy with effusion; and it was his custom now to aspirate as soon as he found sufficient fluid to give the physical signs near the angle of the scapula. Since adopting this method he had been surprised at the rapidity of recovery. Dr. J. E. Winters indorsed Dr. Delafield's views, as did also Dr. Beverly Robinson. The latter said that he made it a rule to resort to aspiration early in all his hospital cases during the past ten or fifteen years, and had certainly seen no harm come from this frequent use of the aspirator; nevertheless, he did not believe that a single aspiration would cure the average case of acute pleurisy with effusion. Dr. Henry P. Loomis called attention to the interesting fact that pulmonary tuberculosis was sometimes promptly arrested by the occurrence of a pleurisy with effusion.

A part of the "A B C" of surgery is the treatment of ulcers, yet we find considerable diversity of opinion regarding the best methods, and a tendency to shift the responsibility (and blame) upon the junior surgical dresser. The writer well remembers hearing that forceful and enthusiastic clinical teacher, the late Professor James L. Little, endeavor to impress upon the minds of his students the importance of carefully attending to the treatment of "only an ulcer." He stated that it was the healing of an obstinate ulcer that had given him his first great help in private practice. A method of treating ulcers which has met with much favor from those surgeons who have tried it is familiarly known here as "Powell's method." It was devised by Dr. Seneca D. Powell, but, so far as I know, it has not been published in any text-book. To the superficial observer it may seem to be only a modification of the well known "basket strapping"; but such is not the case, as its object is not at all to endeavor to approximate the edges of the ulcer. After the ulcer and adjacent parts have been thoroughly cleansed with strong soap and solution of carbolic acid, the dressing is applied, provided always there is a fairly healthy granulating surface. If the ulcer is foul and covered with a deep slough, this slough is removed, as usual, by curetting, and the excavation filled with a wet carbolized gauze dressing. As soon as a granulating surface is obtained, it is covered by the application of narrow strips of diachylon plaster, *applied vertically*. If applied horizontally, the edges of the plaster strips act as a sort of shelf, retaining the discharges, and so materially interfering with the process of repair. Of course great care is taken to give proper support to the whole limb by the application of a good roller bandage of muslin. Where it seems desirable to make very strong compression, it is better to apply the first bandage with moderate pressure, and then apply over it a second one more snugly. Another practical point made by Dr. Powell is that healing of the ulcer can be greatly expedited if the surgeon carefully removes the thin pellicle which often forms over the ulcer. By this pellicle is not meant the tough leathery mass which sometimes forms the base of an ulcer, but a very thin covering which makes the granulations appear blue instead of red. This pellicle should be gently removed by sliding under it the flat end of a probe. It is probably due to necrosis of the upper layers of the granulations. Dr. Robert A. Sands, who has tested the "Powell method" very carefully, speaks enthusiastically of it, and claims that it yields excellent results even in syphilitic ulcers.

At a recent meeting of the Surgical Section, Dr. G. E. Brewer presented a man upon whom he had performed a Bassini operation sixteen months previously. The interest in this case centered on the enormous size of the hernia. It measured a little less than two feet in circumference, and contained eleven feet of intestine and two pounds of omentum. Seven inches of the intestine were so adherent to the bottom of the sac as to be separated with difficulty. The man was kept in bed for six weeks after the operation. Notwithstanding the great size of this hernia, there has been no recurrence since the operation.

All who have had occasion to preserve gross specimens for museum purposes have felt the need of some method by which the natural color would be preserved. Several different modes of procedure have been advocated, one of the latest being that described before the New York Pathological Society by Dr. John H. Larkin. He dries the fresh specimens in the air for an hour or so, and after allowing them to soak in a two to five per cent. solution of formalin for several days, preserves them permanently in 95 per cent. alcohol.

OGDEN C. LUDLOW, M.D.

*2309 Seventh Avenue.*

